# Predictive value of circulating fibroblast growth factor-23 and Klotho on protein-energy wasting in patients undergoing hemodialysis

**DOI:** 10.3389/fnut.2024.1497869

**Published:** 2025-01-07

**Authors:** Xiaoling Zhou, Yang Luo, Yidan Guo, Meng Jia, Chunxia Zhang, Zhihua Shi, Ye Du

**Affiliations:** ^1^Department of Nephrology, Beijing Shijitan Hospital, Capital Medical University, Beijing, China; ^2^Department of Medical Oncology, Beijing Chest Hospital, Capital Medical University and Beijing Tuberculosis and Tumor Research Institute, Beijing, China

**Keywords:** hemodialysis, protein-energy wasting, fibroblast growth factor-23, Klotho, malnutrition

## Abstract

**Background:**

As a state of metabolic and nutritional derangements, protein-energy wasting (PEW) is highly prevalent and associated with increased morbidity and mortality in hemodialysis patients. Fibroblast growth factor-23 (FGF-23) and Klotho have been proven to contribute to chronic kidney disease-mineral and bone disorder (CKD-MBD) in patients undergoing hemodialysis. Previous evidence suggested that FGF-23 and Klotho may also contribute to the malnutritional status among these patients; however, the inter-relationship between the FGF-23–Klotho axis and PEW remains unclear. Therefore, we conducted this cross-sectional study to evaluate the association between plasma FGF-23 and Klotho levels and PEW in hemodialysis patients and to explore whether these markers could predict the presence of PEW.

**Methods:**

Plasma concentrations of FGF-23 and Klotho were measured, and their associations with PEW were assessed. PEW was evaluated based on body weight, muscle mass, biochemical data, and protein and energy intake, according to the 2008 criteria from the International Society of Renal Nutrition and Metabolism (ISRNM).

**Results:**

In this study, 147 hemodialysis patients (mean age 61.05 ± 13.32 years) were enrolled, of whom 66 (44.90%) had PEW. PEW was significant positively correlated with FGF-23 (*r* = 0.403, *p* < 0.001), age (*r* = 0.225, *p* = 0.006), C-reactive protein (*r* = 0.236, *p* = 0.004), intact parathyroid hormone (*r* = 0.237, *p* = 0.004), and single-pool Kt/V (*r* = 0.170, *p* = 0.040), while it was negatively correlated with Klotho (*r* = −0.361, *p* < 0.001), hemoglobin (*r* = −0.215, *p* = 0.009), and serum creatinine (*r* = −0.278, *p* = 0.001). Logistic regression analyses showed that plasma FGF-23 and Klotho were independently associated with PEW, even after adjusting for covariables. The area under the ROC curve (AUC) of FGF-23 and Klotho in predicting PEW was 0.734 and 0.710 (*p* < 0.001), respectively. When the combination of FGF-23 and Klotho was used to predict PEW, its sensitivity was 81.8%, specificity was 60.5%, and the AUC was 0.746.

**Conclusion:**

Plasma levels of FGF-23 and Klotho are associated with PEW in hemodialysis patients. Higher plasma FGF-23 levels and lower Klotho levels may serve as valuable predictors of PEW in these patients.

## 1 Introduction

As a state of metabolic and nutritional derangements, protein-energy wasting (PEW) in patients with chronic kidney disease (CKD) has received great attention in recent years, not only because it has high prevalence among these patients but also because the presence and severity of PEW are closely related to morbidity and mortality among patients with all stages of CKD (1, 2). Previous studies suggested that PEW symptoms were observed in almost 20% of stage 3–5 non-dialysis CKD patients, and the prevalence sharply increased to more than 55% once the patients initiated maintenance dialysis (3). Facing such a serious challenge, it is crucial to identify PEW as early as possible in patients undergoing hemodialysis; however, the current applied diagnostic criteria created by the International Society of Renal Nutrition and Metabolism (ISRNM) require a number of variables to be known and physical examinations to be performed; the complexity of this criteria limits its practical application in clinical settings. So finding more concise predictors of PEW in CKD patients seems to be necessary (4).

The fibroblast growth factor-23 (FGF-23)–Klotho axis plays a major role in chronic kidney disease-mineral and bone disorder (CKD-MBD) in patients with CKD (5, 6). FGF-23 is secreted mainly by osteocytes, and its plasma levels increase 100- to 1,000-fold higher in the end stage of CKD (7, 8). The etiology of plasma-elevated FGF-23 in CKD is mostly because of decreased renal clearance and a feedback response to elevated serum phosphate, and FGF-23 has a feedback relationship with its co-receptor Klotho (9, 10). Studies have shown that plasma Klotho levels decrease during the early stage of CKD (11). Klotho protein exerts diverse effects on the physiological regulation of serum calcium, phosphate, and energy metabolism by influencing the endocrine activities of the FGF-23 (12, 13). In addition to these events, accumulating evidence has shown that plasma FGF-23 and Klotho are associated with vascular calcification, infection, anemia, systemic inflammation, and oxidative stress in hemodialysis patients, which are commonly observed and closely associated with the presence and severity of PEW in hemodialysis patients (14–17). However, the role of plasma FGF-23 and Klotho in metabolic and nutritional status remains unclear, and literature on the relationship between circulating FGF-23–Klotho levels and PEW in hemodialysis patients is scarce (3).

Therefore, we conducted a cross-sectional study to evaluate the relationship between plasma FGF-23 and Klotho and PEW in hemodialysis patients and to explore whether plasma FGF-23 and Klotho could predict the presence of PEW in these patients.

## 2 Methods

### 2.1 Study design and population

We conducted a cross-sectional study on patients who underwent hemodialysis at the Dialysis Center of Beijing Shijitan Hospital, Capital Medical University from June 2023 to January 2024. The inclusion criteria were as follows: (1) patients with end-stage renal disease (ESRD) undergoing hemodialysis for at least three consecutive months and (2) age ≥ 18 years. The exclusion criteria were: (1) acute kidney injury, (2) merge malignant tumors and chronic wasting diseases, (3) severe infections, heart failure, and gastrointestinal bleeding in the past three months, (4) long-term use of glucocorticoids and immunosuppressants, and (5) unable to cooperate with the examiner for various reasons. All patients were prescribed routine hemodialysis three times a week for 4–5 h each time, with a blood flow rate of 200–250 mL/min and a dialysate flow rate of 500 mL/min.

The study was approved by the Ethics Committee of Beijing Shijitan Hospital [sjtky11-1x-2022(079)]. The protocol complied with the Declaration of Helsinki; all participants provided written consent.

### 2.2 Data collection and laboratory measurements

We collected participants’ data, including gender, age, etiology of ESRD, comorbidities, medications used, dialysis access, and dialysis vintage (months). Blood samples were obtained before and after the first dialysis session of the week, following a two-day interval. All blood samples were centrifuged and stored at −80°C. The hemoglobin, urea nitrogen, creatinine, albumin, prealbumin, cholesterol, triglyceride, calcium, phosphate, intact parathyroid hormone (iPTH), and C-reactive protein (CRP) levels were measured using standard laboratory techniques with an auto-analyzer. The plasma C-terminal FGF-23 and Klotho levels were measured using enzyme-linked immunoassays (human FGF-23 ELISA kit: Immutopics, San Clemente, CA, USA, human Klotho ELISA kit: Cusabio, China). Urea clearance index single-pool Kt/V (sp. Kt/V) was calculated from the pre- and post-dialysis serum urea nitrogen levels using the Daugirdas formula (18).

### 2.3 Dietary intake

The lead research dietitian guided participants in filling out their dietary records. Averages were then computed from these three-day records for total energy (kcal and kcal/kg) and total protein (g and g/kg).

### 2.4 Physical measurements

Body mass index (BMI, kg/m^2^) was calculated by dividing the dry weight (kg) by the square of the height (m). Mid-upper arm circumference (MUAC) reflects a patient’s muscle and fat mass. Measurements were conducted using a plastic tape at the midpoint of the arm, avoiding vascular access. The midpoint of the arm was located halfway between the shoulder tip and the elbow tip. Triceps skinfold (TSF) was measured with a skinfold caliper with the arm hanging relaxed at the side. The measurements were repeated twice, and the average value was used for analysis. The mid-upper arm muscle circumference (MUAMC) was calculated by the formula: MUAMC (cm) = MUAC (cm) – 3.14 × TSF (cm). These values were compared to the reference values provided by Frisancho (19).

### 2.5 Handgrip strength measurement

The patients underwent three tests while seated, with the arm positioned alongside the trunk, hips, and knees flexed at 90°, and the wrist pronated. Hand grip strength (HGS) was defined as the average measurement and assessed in the arm opposite the arteriovenous fistula.

### 2.6 Protein-energy wasting evaluation

We evaluated the presence of PEW for each patient based on the criteria outlined by ISRNM in 2008 (20). PEW was considered present if at least three of the following four criteria were met: (1) serum albumin <3.8 g/100 mL or serum prealbumin <30 mg/100 mL, (2) BMI < 23 kg/m^2^, (3) reduced MUAMC (> 10% compared with the 50th percentile of the reference population), (4) dietary protein intake <0.8 g/kg/day.

### 2.7 Statistical analysis

Descriptive data were presented as frequency counts and proportions for categorical variables, means ± standard deviations (SDs) for normally distributed continuous variables, and medians with interquartile ranges (IQRs) for skewed variables. The comparison of variables between the two PEW groups used chi-squared tests, T-tests, or Mann–Whitney U tests, as they were appropriate. The correlation between PEW and clinical variables was examined using Spearman rank correlation analysis. Univariable and multivariable logistic regression analyses were conducted to assess the influence of the plasma FGF-23 and Klotho levels on the risk of PEW, with PEW as the dependent variable, plasma FGF-23 or Klotho as the independent variables entered into the multivariate logistic regression models (model 0: unadjusted; model 1: adjusted for age, gender; model 2: adjusted for age, gender, and all covariates with a *p* value of less than 0.05 on the univariable analysis). To further evaluate the predictive power of the plasma FGF-23 and Klotho for PEW against the ISRNM standards, a receiver operating characteristic (ROC) curve analysis was performed. The area under the curve, sensitivity, and specificity of the plasma FGF-23 and Klotho at various cutoffs were calculated. Optimal cut-off points were determined using the maximum value of Youden’s index (calculated by sensitivity + specificity – 1). The predictive value of plasma FGF-23 and Klotho concentrations on PEW was evaluated by calculating the area under the ROC curve (AUC). All the analyses were performed with SPSS version 29.0 statistical software (SPSS Inc., Chicago, IL, USA). Statistical significance was set at a value of *p* < 0.05.

## 3 Results

### 3.1 Characteristics of participants

We included 147 hemodialysis patients (including 48 females) with an age of 61.05 ± 13.32 years (21–85 years) and the median dialysis vintage of 61 (37, 115) months. The etiologies of the included patients were as follows: diabetes (37.41%), glomerulonephritis (35.37%), hypertension (13.61%), polycystic kidney disease (4.76%), and others (8.84%).

Among them, there were 66 patients (44.90%) with PEW. PEW subjects tend to be older, and with higher levels of iPTH, CRP, FGF-23, and sp. Kt/V, and lower levels of hemoglobin, triglycerides, serum creatinine, and Klotho (all *p* < 0.05) (Table 1). The characteristics of the components of PEW are shown in Table 2.

**Table 1:** Clinical characteristics of hemodialysis patients according to PEW status.

Characteristics	Total (*n* = 147)	PEW group (*n* = 66)	Non-PEW group (*n* = 81)	*p*-value
Age (years)	61.05 ± 13.32	64.29 ± 13.45	58.42 ± 12.70	0.007
Male sex, *n* (%)	99 (67.35)	45 (68.2)	54 (66.67)	0.846
Smoking, *n* (%)	48 (32.65)	20 (30.30)	28 (34.57)	0.583
Diabetes mellitus, *n* (%)	84 (57.14)	41 (62.12)	43 (53.09)	0.271
Hypertension, *n* (%)	121 (82.31)	56 (84.85)	65 (80.24)	0.467
Medication use, *n* (%)				
Phosphate binders	97 (65.98)	46 (69.70)	51 (62.97)	0.391
Vitamin D	47 (31.97)	23 (34.85)	24 (29.63)	0.500
Cinacalcet	18 (12.24)	8 (12.12)	10 (12.35)	0.967
Dialysis access, *n* (%)				
Arteriovenous fistula	112 (76.19)	48 (72.73)	64 (79.01)	0.374
Central venous catheter	35 (23.81)	18 (27.27)	17 (20.99)	0.374
Dialysis vintage (months)	61.00 (37.00–115.00)	49.00 (35.75–115.00)	66.00 (38.50–118.00)	0.356
sp. Kt/V	1.21 ± 0.27	1.27 ± 0.25	1.15 ± 0.27	0.007
Laboratory parameters				
Hemoglobin (g/L)	113.73 ± 12.74	110.18 ± 12.46	116.63 ± 12.29	0.002
Triglycerides (mmol/L)	1.65 ± 1.02	1.35 ± 0.91	1.88 ± 1.04	0.001
Total cholesterol (mmol/L)	3.45 ± 0.89	3.33 ± 0.94	3.54 ± 0.84	0.153
Serum creatinine (μmol/L)	880.00 (721.00–1093.00)	826.00 (660.50–972.50)	965.00 (764.50–1243.50)	0.010
Calcium (mmol/L)	2.09 ± 0.68	2.06 ± 0.69	2.12 ± 0.68	0.559
Phosphorus (mmol/L)	2.14 ± 0.20	2.11 ± 0.22	2.17 ± 0.18	0.064
iPTH (pg/mL)	287.70 (162.50–458.40)	313.30 (224.35–504.68)	240.50 (123.10–404.00)	0.004
CRP (mg/L)	5.18 (2.04–11.56)	9.46 (2.93–14.20)	4.26 (1.78–8.18)	0.004
FGF-23 (RU/L)	23.02 (17.88–31.32)	28.28 (22.08–35.06)	20.07 (14.91–24.81)	<0.001
Klotho (RU/L)	19.33 (12.96–25.91)	15.62 (11.35–22.54)	23.02 (16.04–32.95)	<0.001

Quantitative data are shown as means ± standard deviations or medians (interquartile range) according to normality. Qualitative data are shown as numbers (percentages). Chi-squared tests were used for qualitative variables. T-tests or Mann–Whitney U tests were used to compare measurements between PEW and non-PEW groups according to normality. PEW, protein-energy wasting; sp. Kt/V, single-pool Kt/V; iPTH, intact parathyroid hormone; CRP, C-reactive protein; FGF-23, fibroblast growth factor-23.

**Table 2:** Characteristics of the components of PEW.

Characteristics	Total (*n* = 147)	PEW group (*n* = 66)	No-PEW group (*n* = 81)	*p*-value
Prealbumin (g/L)	287.20 ± 84.56	270.77 ± 86.03	300.58 ± 81.43	0.033
Albumin (g/L)	36.46 ± 3.67	35.60 ± 3.98	37.16 ± 3.25	0.010
DPI (g/kg/day)	0.76 ± 0.12	0.70 ± 0.13	0.80 ± 0.09	<0.001
BMI (kg/m^2^)	23.29 ± 4.25	20.75 ± 2.94	25.36 ± 4.03	<0.001
MUAC (cm)	26.83 ± 3.94	25.02 ± 3.87	28.30 ± 3.35	<0.001
MUAMC (cm)	20.99 ± 3.14	20.40 ± 3.54	21.47 ± 2.71	0.040
TSF (cm)	1.88 ± 0.77	1.48 ± 0.59	2.20 ± 0.75	<0.001
HGS (Kg)	22.29 ± 7.37	18.32 ± 5.15	25.53 ± 7.33	<0.001

Quantitative data are shown as means ± standard deviation. T-tests were used to compare measurements between PEW and no-PEW groups. PEW, protein-energy wasting; DPI, dietary protein intake; BMI, body mass index; MUAC, mid-upper arm circumference; MUAMC, Mid-upper arm muscle circumference; TSF, triceps skinfold; HGS, handgrip strength.

### 3.2 Correlations between PEW and clinical parameters

Significant positive correlations were observed between PEW and FGF-23, age, CRP, iPTH, and sp. Kt/V. Conversely, PEW was negatively correlated with Klotho, hemoglobin, and serum creatinine (Table 3).

**Table 3:** Correlations between PEW and clinical variables.

Variables	Correlation coefficient	*p*-value
Age (years)	0.225	0.006
Hemoglobin (g/L)	−0.215	0.009
Serum creatinine (μmol/L)	−0.278	0.001
CRP (mg/L)	0.236	0.004
iPTH (pg/mL)	0.237	0.004
FGF-23 (RU/L)	0.403	<0.001
Klotho (RU/L)	−0.361	<0.001
sp. Kt/V	0.170	0.040

PEW, protein-energy wasting; CRP, C-reactive protein; iPTH, intact parathyroid hormone; FGF-23, fibroblast growth factor-23; sp. Kt/V, single-pool Kt/V.

### 3.3 Associations between the levels of plasma FGF-23 and Klotho and PEW

The associations between the levels of plasma FGF-23 and Klotho with PEW are presented in Table 4. The level of plasma FGF-23 (OR: 1.087; 95% CI: 1.044–1.132) and Klotho (OR: 0.951; 95% CI: 0.919–0.983) were significantly associated with PEW in unadjusted analyses. After further multivariate adjustment (Model 1 and 2), these associations remained statistically significant.

**Table 4:** Logistic regression analyses for the association between the levels of plasma FGF-23 and Klotho and PEW.

Models	FGF-23 (RU/L)		Klotho (RU/L)	
	OR (95% CI)	*p*-value	OR (95% CI)	*p*-value
Model 0	1.087 (1.044–1.132)	<0.001	0.951 (0.919–0.983)	0.003
Model 1	1.089 (1.044–1.137)	<0.001	0.952 (0.919–0.986)	0.006
Model 2	1.091 (1.042–1.141)	<0.001	0.960 (0.926–0.995)	0.027

Model 0: unadjusted; Model 1: adjusted for age, gender; Model 2: adjusted for age, gender, sp. Kt/V, hemoglobin, triglycerides, serum creatinine, iPTH and CRP. FGF-23, fibroblast growth factor-23; OR, odds ratio.

### 3.4 ROC curve analysis

**Figure 1:**
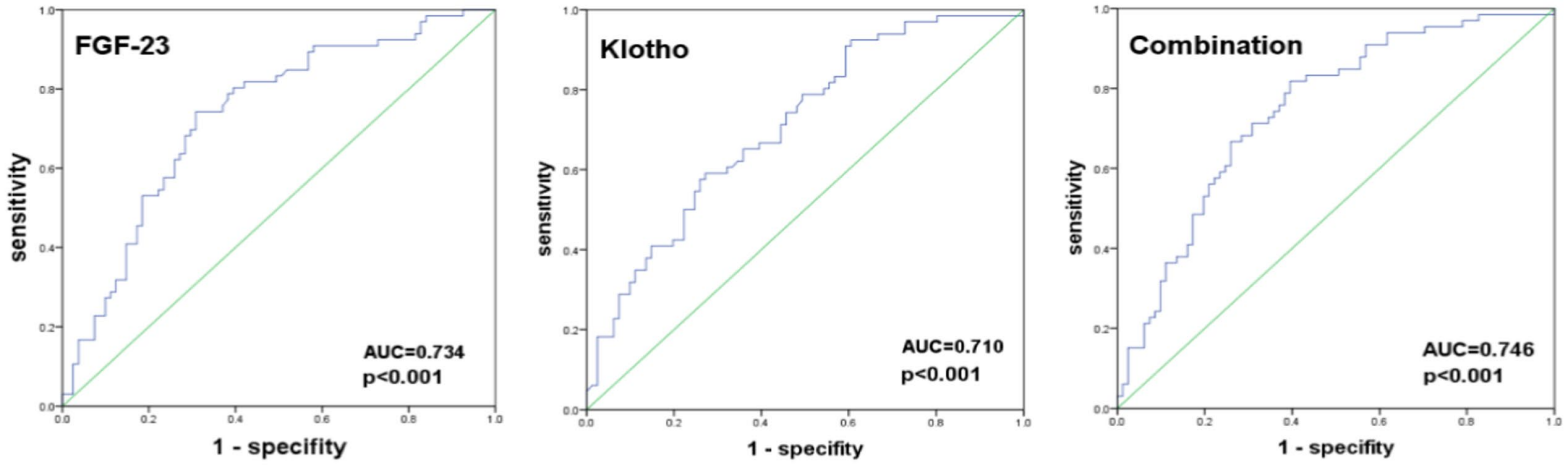
ROC curves for predicting the probability of PEW.

The ROC analysis identified an optimal cut-off for FGF-23 at 22.97 RU/L, with a sensitivity of 74.2%, a specificity of 69.1%, and an area under the curve (AUC) of 0.734 (95% CI: 0.652–0.815); an optimal cut-off for Klotho at 17.61 RU/L, with a sensitivity of 59.1%, a specificity of 72.8%, and an area under the curve (AUC) of 0.710 (95% CI: 0.627–0.792). When the combination of FGF-23 and Klotho was used to predict PEW, its sensitivity was 81.8%, specificity was 60.5%, and area under the curve (AUC) was 0.746 (95% CI: 0.667–0.826) (Figure 1; Table 5).

**Table 5:** Evaluation of the efficacy of FGF-23 and Klotho for predicting PEW.

Variables	AUC (95% CI)	Cut-off value	Sensitivity (%)	Specificity (%)	*p*-value
FGF-23 (RU/L)	0.734 (0.652–0.815)	22.97	74.2	69.1	<0.001
Klotho (RU/L)	0.710 (0.627–0.792)	17.61	59.1	72.8	<0.001
Combination	0.746 (0.667–0.826)	-	81.8	60.5	<0.001

PEW, protein-energy wasting; FGF-23, fibroblast growth factor-23.

## 4 Discussion

In this cross-sectional study, we found that 44.9% of hemodialysis patients experienced PEW. Significant factors such as increased age, sp. Kt/V, CRP, iPTH, and decreased hemoglobin and serum creatinine were associated with PEW in these patients. Additionally, higher plasma FGF-23 and lower plasma Klotho concentrations were independently associated with PEW, even after adjusting for confounding factors. ROC analysis indicated that plasma FGF-23 and Klotho levels may serve as useful predictors for identifying PEW in hemodialysis patients.

PEW is a common metabolic and nutritional disorder in patients undergoing hemodialysis (21, 22). Depending on the 2008 ISRNM diagnostic criteria, the prevalence of PEW ranged from 30 to 75% in hemodialysis patients (23). In the present study, nearly half of the hemodialysis patients were affected by PEW. Previous studies have shown that PEW in CKD is quite different from that in the general population, encompassing a broader scope of pathologies related to poor nutritional status in the uremic milieu, persistent inflammation, increased oxidative stress, and other CKD-related complications, such as CKD-MBD and acidemia (24). These factors can decrease anabolism or increase catabolism, which may increase resting energy expenditure and cause hypermetabolism, eventually leading to progressive muscle wasting and frailty (25). In our study, the risk factors that reflect the above-mentioned pathological status in hemodialysis patients were also validated. Furthermore, multiple studies have indicated that PEW is closely associated with major adverse clinical outcomes, including increased hospitalization and mortality (26). Thus, early identification of PEW is quite important. However, the currently recommended criteria by ISRNM are complex and involve assessing multiple variables and subjecting the patient to physical examinations, impeding its practical application in clinical settings. Finding sensitive predictors that can be applied more easily in clinical practice is therefore necessary. FGF-23 and Klotho are well-known for their important role in CKD-MBD. Recently, increasing evidence suggests that FGF-23 and Klotho are associated with systemic inflammation and oxidative stress in hemodialysis patients. These results gave us a hint that FGF-23 and Klotho might have a potential relationship with PEW. Investigation of the association between the FGF-23–Klotho axis and PEW may provide new insights into early identification and prevention of PEW.

Several previous studies have focused on the correlation between plasma FGF-23 levels and the metabolic and nutritional status of hemodialysis patients. Montford et al. assessed the associations of plasma FGF23 concentrations with BMI, total cholesterol, low-density lipoprotein-cholesterol, high-density lipoprotein-cholesterol, and triglycerides (27). These results indicated that higher plasma FGF23 levels are associated with lower BMI and blood lipid levels in dialysis patients. Decreased BMI is one of the PEW diagnostic criteria, and has been shown to correlate with poor outcomes in patients requiring chronic dialysis. Montford et al.’s study seems consistent with our study, suggesting a link between FGF-23 and the nutritional status of hemodialysis patients. In another study by Fukasawa et al., the authors assessed the relationship between the FGF-23–Klotho system and muscle mass and found that FGF-23 was an independent predictor of muscle mass (28). These results seem somewhat different from our study. This discrepancy may be due to differences in study populations and nutritional assessment indicators. Fukasawa et al. used creatinine production rate and abdominal muscle area as muscle mass indicators. Creatinine production rate may be influenced by residual renal function and dialysis dose, while abdominal muscle area was related to gender and measurement method.

The FGF23–Klotho axis plays an important role in CKD-MBD and is involved in mineral metabolism. In the present study, we controlled for phosphorus, calcium, and parathyroid hormone, but FGF-23 and Klotho were still independently associated with PEW. There may be some interconnections between FGF23–Klotho and known mechanisms of PEW in the setting of CKD. A cross-sectional study of patients receiving maintenance hemodialysis demonstrated that patients in the highest tertile group stratified by serum FGF23 levels had a higher odds ratio for increased serum inflammatory markers (29). Other previous studies have shown that FGF-23 can directly stimulate the production of proinflammatory cytokines (via FGFR4 binding and activation on hepatocytes) and amplify systemic inflammation (14, 30) while interleukin-6 and tumor necrosis factor-*α* have been verified to promote muscle protein degradation and muscle wasting (31). In addition, previous studies have shown a positive association between FGF-23 and insulin resistance, which can decrease insulin-stimulated protein synthesis and increase protein degradation in skeletal muscle, and may play a key role in PEW (32, 33). On the other hand, Klotho has been shown to have anti-inflammatory and anti-oxidative stress effects (34, 35). Several *in vivo* studies suggest that Klotho may play a role in carbohydrate metabolism (36). Collectively, these data suggest the interplay between FGF23–Klotho and energy metabolism, although the mechanism remains to be elucidated.

Given the complexity of the ISRNM diagnostic criteria for PEW, we also explored the predictive value of plasma FGF-23 and Klotho using ROC curve analysis. Our findings indicated that plasma FGF-23 and Klotho levels could serve as concise predictors of PEW in hemodialysis patients. However, these results still need to be validated in future prospective clinical studies.

Although we have provided data on the association between FGF-23–Klotho and PEW, as well as identifying their predictive value of PEW, some limitations must be mentioned. First, due to the cross-sectional study design, a longitudinal causal relationship cannot be established between the changes in the plasma FGF-23–Klotho levels and alterations in PEW. Second, as a single-center study, selection bias may have been introduced; therefore, caution must be exercised before extrapolating our results to other hemodialysis patients. Larger sample sizes and validation among different cohorts should be needed to confirm our results. Third, the cut-off values used in our study should be carefully extrapolated to other patients, given the differences in research cohorts and detection methods. Fourth, we did not measure vitamin D levels in this study population, although one of the primary physiological actions of FGF-23 is vitamin D metabolism, and the association between vitamin D levels and muscle mass has been reported.

## 5 Conclusion

The present study found that plasma FGF-23 and Klotho levels are associated with PEW in hemodialysis patients. Higher plasma FGF-23 and lower Klotho levels may be valuable predictors of PEW in hemodialysis patients. Thus, regular assessment of plasma FGF-23 and Klotho in patients on hemodialysis may help predict the existence of PEW, and this could facilitate timely intervention of this pathological status.

## Data Availability

The original contributions presented in the study are included in the article/supplementary material, further inquiries can be directed to the corresponding author.
